# Periventricular cyst as a complication of ventriculoperitoneal shunting in the context of intracranial haemorrhage: a case report and review of the literature

**DOI:** 10.1093/jscr/rjad743

**Published:** 2024-01-23

**Authors:** Leon E Smith, Brian D Zeman

**Affiliations:** Department of Rehabilitation Medicine, Royal North Shore Hospital, Reserve Road St Leonard's 2065, NSW, Australia; Department of Rehabilitation Medicine, Royal North Shore Hospital, Reserve Road St Leonard's 2065, NSW, Australia

**Keywords:** ventriculoperitoneal shunt, pericatheter cyst, hydrocephalus, shunt revision

## Abstract

Spontaneous intraventricular haemorrhage with hydrocephalus frequently requires neurosurgical intervention, including ventriculoperitoneal shunting. We describe a periventricular cyst following the placement of a ventriculoperitoneal shunt in a 67-year-old female patient. The patient was admitted for rehabilitation after a spontaneous left basal ganglia and diffuse intraventricular haemorrhage with hydrocephalus. Initial management included an extraventricular drain, followed by a ventriculoperitoneal shunt. On Day 5 of rehabilitation, the patient was urgently reviewed for reduced level of consciousness. A cerebrospinal fluid cyst was identified around the shunt catheter, with subacute haemorrhage within the cyst. The patient underwent a successful shunt revision, with rapid improvement in consciousness and resolution of the cyst. This case highlights the importance of pericatheter cyst as a differential diagnosis in patients with altered neurological status following ventriculoperitoneal shunting. Early detection and surgical revision can lead to rapid resolution of symptoms and a favourable prognosis.

## Introduction

Ventriculoperitoneal shunting is commonly performed as a treatment for hydrocephalus, serving to divert excess cerebrospinal fluid (CSF) to the peritoneal cavity. Although effective, complications are not uncommon, with shunt revision rates in the literature being reported at 20–30% [1, 2]. Proximal shunt obstruction, which can cause recurrent hydrocephalus and raised intracranial pressure, is among the most common complications requiring revision surgery [2]. Pericatheter cyst formation is a rare complication of proximal shunt failure, wherein a localized collection of CSF is detected in close proximity to the catheter. This can present with focal neurological symptoms as well as nausea, headache and altered consciousness [3]. We report on a case of pericatheter cyst formation in a 67 year old woman, with a favourable outcome with revision surgery.

## Case presentation

**Figure 1:**
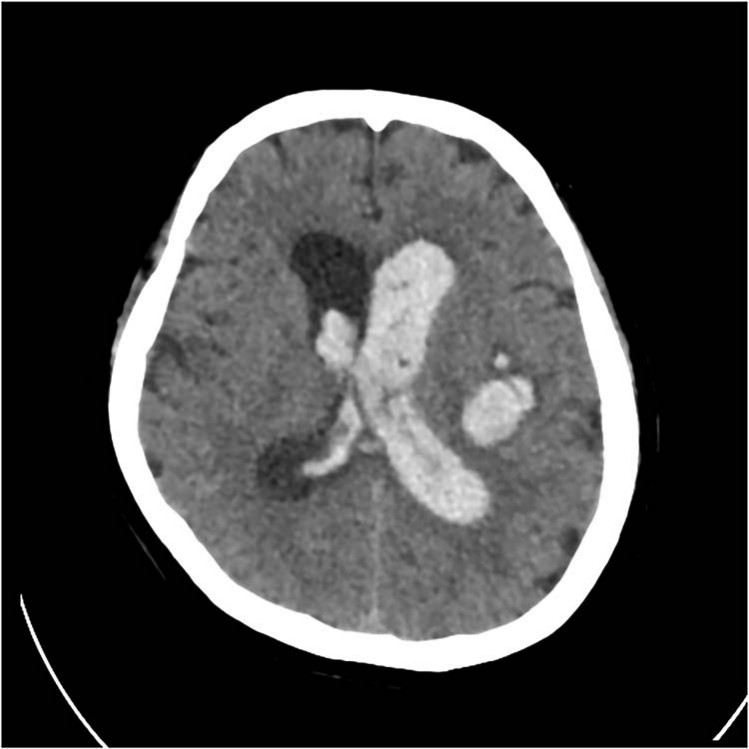
Initial CT revealing acute left-sided intraparenchymal haemorrhage with intraventricular extension and mass effect.

A 67 year-old woman was admitted to our service from the neurosurgical department for rehabilitation after a spontaneous left basal ganglia haemorrhage and diffuse intraventricular haemorrhage with hydrocephalus. She had been initially found unresponsive at home with coffee ground vomitus and incontinence of urine. An urgent computed tomography (CT) scan of the brain showed a large intracranial haemorrhage with intraventricular bleeding and hydrocephalus (Fig. 1). Her acute admission involved an extraventricular drain inserted by the neurosurgery service. A repeat CT brain performed 10 days later showed stable multicompartmental haemorrhage, and the extraventricular drain was removed. A ventriculoperitoneal shunt was inserted on Day 27 for ongoing management of the hydrocephalus. A digital subtraction angiogram showed no underlying aneurysm or arteriovenous shunt.

Her previous medical history included only untreated hypertension. She was commenced on amlodipine and metoprolol (the latter being commenced due to sinus tachycardia). Prior to admission she was fully independent and driving. Upon admission to rehabilitation, she was reliant on nasogastric feeds and had right hemiparesis with limited use of the right upper limb, and no movement in the right lower limb.

**Figure 2:**
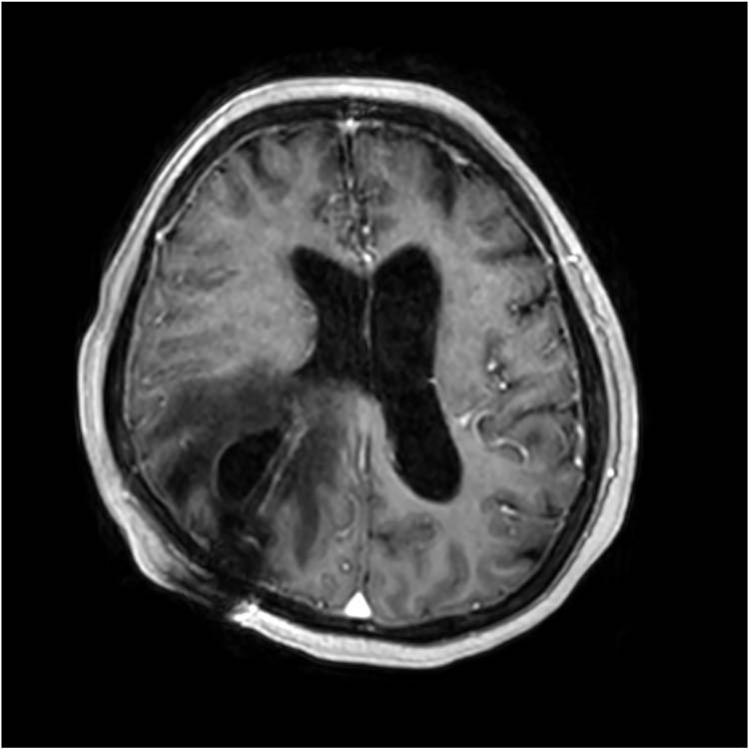
Contrast-enhanced T1-weighted MRI scan showing presence of the intraparenchymal cyst and surrounding vasogenic oedema.

On Day 5 of her rehabilitation admission (29 days after the shunt was inserted), a rapid response call was initiated for reduced level of consciousness (Glasgow coma scale 3). An urgent CT brain was performed which showed an extensive area of low density in the right parietal and posterior frontal lobe adjacent to the entry of the ventricular shunt, with some subarachnoid blood in the adjacent sulci. The patient was transferred back to the acute hospital, where an urgent magnetic resonance imaging (MRI) scan demonstrated a likely CSF cyst around the shunt catheter, with subacute haemorrhage within the cyst (Fig. 2). An abdominal CT scan showed no evidence of distal shunt obstruction. A surgical revision of the ventriculoperitoneal shunt took place the next day. Brisk flow was confirmed in the distal catheter without its Codman® Certas valve connected. A small amount of CSF egress was found around the ventricular catheter, and a replacement proximal catheter was inserted and connected to a new Certas valve.

**Figure 3:**
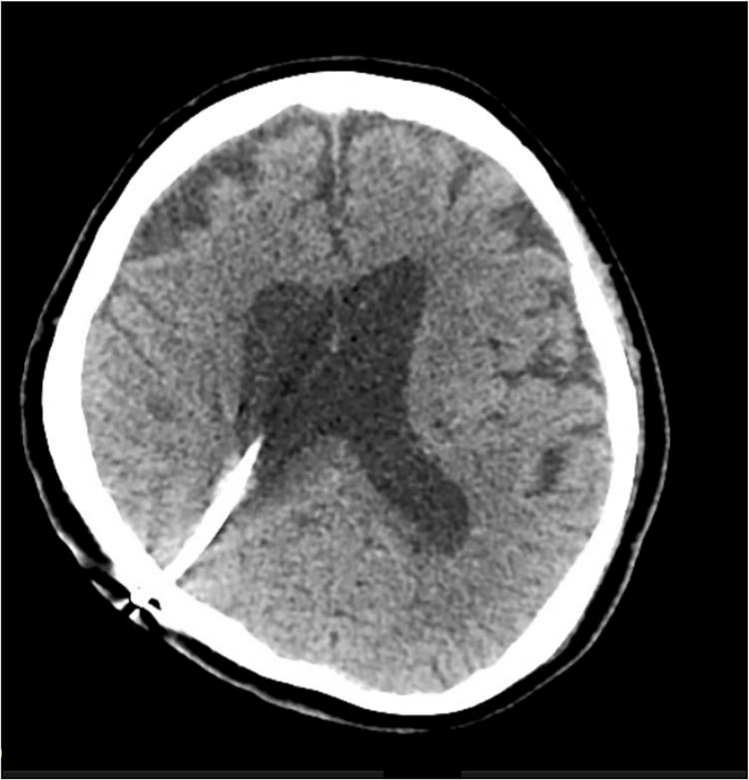
CT scan performed 41 days after shunt revision, showing resolution of the periventricular cyst.

On clinical review after the shunt revision, the patient’s level of consciousness had improved, with her Glasgow Coma Scale returning to 15. She was alert and conversational in both English and her native language, and was moving all limbs (although her pre-existing right hemiparesis persisted). A repeat CT brain was performed 1 day postoperatively which showed resolution of the cyst and reduced midline shift from 4 to 3 mm. An additional CT was performed 8 days after the revision, which revealed further improvement with reduced oedema and almost completes normalization of the midline shift to 1 mm. The patient was subsequently returned to the rehabilitation department for ongoing therapy and discharge planning. A progress CT done 41 days after the shunt revision showed no further evidence of the cyst, although ventricular dilatation persisted (Fig. 3).

Her cognition fluctuated throughout the course of her admission, and despite her initial improvement postoperatively she remained persistently disoriented with poor memory. In view of this, and combined with her other impairments, she was deemed to have high level care needs with an unfavourable functional prognosis. She was ultimately discharged to a residential aged care facility.

## Discussion

Pericatheter cyst is considered an extremely rare complication of ventriculoperitoneal shunting. Although over 30 000 shunts are performed annually within the United States alone [4], less than 35 cases of pericatheter cyst have been reported in the literature. Karydakis *et al.* [3] reviewed 20 published cases, identified as of 2018, and found that the most common presenting symptoms were headache (30% of cases) and vomiting (25% of cases). The time from shunt placement to the formation of a pericatheter cyst can be anything from 1 week to more than 15 years [3].

Formation of the cyst appears to result from raised CSF pressure in the ventricular system, causing egress of the CSF into surrounding tissue. The formation of a distinct cyst, as opposed to diffuse oedema, appears to result from a combination of predisposing factors, including rapid increase of pressure and formation of gliosis around the catheter [5]. Malpositioning of the catheter, such that catheter fenestrations are located outside the ventricle, may also predispose towards cyst formation [5, 6].

Findings on imaging can include mass effect and oedema on CT and can mimic the appearance of a tumour or abscess [7, 8]. A low index of suspicion for cyst formation, which is unsurprising given its rarity, may result in misdiagnosis and ineffective or unnecessary treatment. If an abscess is suspected, for instance, this would usually prompt treatment with empirical antibiotic therapy, particularly if ring enhancement is visible on CT [9]. Once the presence of a pericatheter cyst is confirmed, the usual management approach is revision of the shunt and correction of the obstruction. This usually results in rapid resolution of symptoms and shrinkage or complete resolution of the cyst, as was the case in our patient.

As of 2023, 31 cases are published in the literature, including this case [5–20]. We reviewed these cases, with relevant symptoms, findings and management details described in Table 1. Including our case, 58% of reported cases are male and 42% were under 18 years of age at the time of presentation. The single most common presenting symptom was headache (14 patients, 45%), followed by nausea/vomiting, (8 patients, 26%). A total of 26 patients ultimately went shunt revision, 2 had the shunt removed entirely, 1 had the cyst aspirated without revision, and 2 were not treated. The vast majority of those for which outcome information was available had a favourable outcome. Only four cases had documented concern for infection or tumour, suggesting that specific imaging findings such as signal isointensity with CSF and lack of contrast enhancement can be used in practice to differentiate periventricular cyst from the more typical findings of tumour and abscess.

**Table 1:** Summary of cases of pericatheter cyst published to date.

Case	Details	Symptoms	Time From Shunt Insertion	Imaging Findings	Concern for Abscess/Tumour	Management/Outcome
Sugimoto *et al.* [10]	Male, 5 month old	Vomiting	2 months	CT: cyst surrounding VP shunt. Nuclear scan: confirmed shunt obstruction.	No	Shunt revised and replaced contralaterally
Sugimoto *et al.* [10]	Female, 2 years old	Vomiting, Reduced consciousness	2 months	CT shuntography: initially showed abdominal obstruction. MRI: confirmed periventricular cyst	No	Repeat shunt revision with resolution
Sugimoto *et al.* [10]	Female, 3 years old	Asymptomatic	2 years	CT: Porencephaly surrounding the shunt catheter	No	Revision x2 required. Resolution of the cyst on follow-up scan
Sugimoto *et al.* [10]	Male, 10 years old	Seizure, Hemiparesis	2 months (Ommaya reservoir)	CT: Porencephaly and intraparenchymal oedema.	No	Removal of chemotherapy reservoir and replacement with VP shunt. Cyst reduced
Sakamoto *et al.* [11]	Male, 7 years old	Subcutaneous fluid collection	3 months	CT: low-density area surrounding the ventricular catheter.	No	Peritoneal component replaced. Symptoms resolved, marginal residual cyst at follow-up
Sakamoto *et al.* [11]	Male, 7 years old	Vomiting, subcutaneous fluid collection	9 months	CT: slit-like lateral ventricles and pericatheter cyst in left frontal lobe.	No	Peritoneal component replaced. Symptoms resolved, marginal residual cyst at follow-up
Sakamoto *et al.* [11]	Male, 19 years old	Headache, Reduced consciousness	1 month	MRI: cystic lesion surrounding ventricular catheter, ending at subcortical white matter	No	Peritoneal component replaced. Symptoms resolved, marginal residual cyst at follow-up
Sakamoto *et al.* [11]	Male, 6 months old	Vomiting, Reduced Consciousness	1 year	CT: cerebrospinal fluid oedema along the catheter in association with periventricular CSF oedema.	No	Peritoneal component replaced. Symptoms and cyst both resolved
Iqbal *et al.* [5]	Male, 10 years old	Headache, Hemiparesis, Vomiting	10 years	CT: hydrocephalus and low-density parenchymal lesion. MRI confirmed presence of cyst.	No	Endoscopic exploration. Removal and replacement of shunt system. Symptoms and limb strength improved
Iqbal *et al.* [5]	Male, 10 years old	Headache Hemiparesis, Nausea	Unknown	CT: hydrocephalus and low density, non-enhancing intraparenchymal lesion surrounding the ventricular catheter in the left frontal region. MRI: pericatheter cystic collection	No	Revision of shunt. Asymptomatic at 6 month follow-up
Vajramani *et al.* [12]	Female, 51 years old	Speech and writing problems	4 years	Serial MRIs showed CSF cyst	No (known tumour separate to the cyst).	Cyst punctured and drained and catheter withdrawn. Cyst reaccumulated thereafter and a new catheter was inserted avoiding the cyst. Cyst and symptoms both completely resolved.
Sinha *et al.* [13]	Male, 4 years old	Ataxia, Gait disturbance	3 years	MRI: pericatheter intraparenchymal cyst	No	Cyst aspirated; shunt not revised. Symptoms resolved over 18 months.
Sinha *et al.* [13]	Male, 5 months old	Asymptomatic	4 weeks	CT: pericatheter cyst.	No	Serial observation scans; cyst slightly improved. No symptoms.
Rim *et al.* [14]	Male, 24 days old	Fontanelle bulging	2 months	Pericatheter cyst reported on CT and MRI	No	Cyst drained via burr-hole. Reaccumulated two-months later and shunt revised. Symptoms resolved
Shekawat *et al.* [15]	Male, 65 years old	Hemiparesis	3 days	MRI: cystic lesions containing CSF around the ventricular catheter with compression of the parietal lobe.	No	Cyst aspirated and VP shunt removed. Hemiparesis resolved.
Amans *et al.* [8]	Male, 20 years old	Headache, Nausea, Hemiparesis, Visual problems	15 years	CT: homogenous low-density lesion in right frontal lobe surrounding the VP shunt. MRI (after shunt removal): multiloculated, spherical, fluid-filled mass with extensive surrounding vasogenic oedema and a thin, mildly T2-hypointense, minimally enhancing rim.	Yes (initially planned for surgical debridement)	Extravencitrular drain inserted. Shunt removed and sent for culture (negative). Contralateral VP shunt inserted. Cyst resolved at follow-up.
Balasubramaniam *et al.* [16]	Male, 18 years old	Seizure, Subgaleal swelling at shunt incision site.	16 months	CT: periventricular porencephalic cyst around VP shunt catheter.	No	Shunt removed entirely. Cyst improved at one month, and seizures resolved.
Watkins *et al.* [17]	Female, 26 years old	Visual problems	1 week	CT: cystic collection around right ventricular catheter. Abdominal x-ray: coiling of right distal catheter. MRI: No ischaemia.	No	Revision of distal catheter with drainage of high-pressure CSF. Visual fields normalised and cyst disappeared on 1 month follow-up imaging.
Bianchi [18]	Male, 19 years old	Hemiparesis	19 years	MRI: right periventricular fronto-parietal cystic lesion surrounding catheter, with oedema. No gadolinium enhancement.	Yes. Planned for cyst excision.	At surgery, intra-ventricular catheter was found to be disconnected with fibrous covering. Upon dissection, high-pressure CSF escape occurred. New shunt was connected to the catheter which was left in place and functioned normally thereafter. Complete resolution of symptoms.
Lam and Gan [6]	Female, 25 years old	Headache, Visual problems	1 month	CT: intraparenchymal cystic lesion isodense with CSF along catheter tract. MRI (post shunt removal): lesion noted to be T1 hypointense and T2 hyperintense with no gadolinium enhancement.	No (CSF culture negative)	Shunt removed and contralateral shunt inserted. Symptoms improved. Cyst regressed significantly by 8 month follow-up
Wallace and Grandhi [19]	Male, 60 years old	Headache, Reduced consciousness, Gait problems	7 weeks	CT: intraparenchymal pericatheter cystic collection with severe oedema. MRI: simple cyst with significant oedema, no restricted diffusion or contrast enhancement of the cyst wall.	No	Shunt revision with proximal catheter replacement. Immediate resolution of symptoms.
Kale *et al.* [7]	Female, 38 years old	Headache	12 months	CT: fluid collection along the tract of the catheter. Nuclear medicine study: no distal obstruction, but focal accumulation in the right frontal fluid collection.	No	Lost to follow-up without any intervention.
Kale *et al.* [7]	Male, 43 years old	Headache	4 years	CT and MR both showed right frontal fluid collection along catheter tract in right frontal lobe. Nuclear medicine study: no transit of CSF, suggesting distal obstruction, and focal uptake suggesting additional proximal obstruction.	No	Shunt revision with rapid improvement of symptoms. Cyst completely resolved at 1-year follow-up
Kale *et al.* [7]	Female, 24 years old	Abdominal pain	5 years	CT: fluid collection and oedema around right frontal shunt. Nuclear medicine study: delayed passage and accumulation in the collection.	No	Revision; outcome not stated.
Kale *et al.* [7]	Female, 43 years old	Headache	3 months	CT and MRI: fluid collection and oedema along left frontal tract. Nuclear medicine study: focal tracer retention in the collection.	No	Revision; outcome not stated.
Kale *et al.* [7]	Female, 46 years old	Headache	2 weeks	CT and MRI: collection and oedema along shunt tract. Nuclear medicine study: no distal obstruction. Focal intracranial accumulation in the region of the fluid collection.	No	Revision; outcome not stated.
Kale *et al.* [7]	Male, 36 years old	Headache, Tinnitus, Visual Problems	15 months	CT and MRI: fluid collection and oedema on shunt tract. Nuclear medicine study: no tracer movement through distal portions of the shunt tubing. Collection of tracer at site of administration.	No	Revision; outcome not stated.
Karydakis *et al.* [3]	Female, 9 years old	Headache Vomiting	4 years	MRI: non-enhancing homogenous cyst in right frontal lobe.	Yes (initially booked for craniotomy)	Shunt revision and cyst drainage. Cyst resolved on subsequent MRI.
Shah *et al.* [9]	Female, 54 years old	Headache, Visual problems	16 years	CT: encephalomalacia with vasogenic oedema. MRI: intraparenchymal cyst in right frontal lobe with surrounding vasogenic oedema with ring enhancement.	Yes (empirical antibiotics commenced)	Burr hole aspiration of cyst and aspiration of shunt reservoir (cultures from both negative). Second shunt placed and distal catheter revised. Cyst unchanged at 4 days. Clinical outcome not stated.
de Oliviera [20]	Female, 22 years old	Headache, Visual problems	3 months	CT: parenchymal lesion around the ventricular catheter with surrounding oedema. MRI: isointense lesion with non-enhancing wall.	No	Shunt revision with replacement of catheter.
Our case	Female, 67 years old	Reduced consciousness	1 month	CT: extensive area of low density right parietal and posterior frontal lobe adjacent to the entry of the ventricular shunt; subarachnoid blood in the adjacent sulci. MRI: likely CSF cyst around the shunt catheter, with subacute haemorrhage within the cyst Abdominal CT: no evidence of distal shunt obstruction.	No	Shunt revision with proximal catheter replacement

This case report not only adds to the limited available literature regarding pericatheter cysts but also highlights the critical role of urgent neuroimaging in identifying shunt complications and guiding appropriate and timely intervention. Early intervention and correction of a pericatheter cyst can facilitate a favourable prognosis, which reversal of symptoms and substantial improvement in the patient’s presentation.

## Conclusion

This case outlines an example of an extremely rare complication of ventriculoperitoneal shunting: the formation of a pericatheter cyst. The clinical presentation of our patient, whose level of consciousness dramatically decreased with the formation of the cyst, emphasizes the importance of early recognition of this complication. The patient’s significant improvement following surgical revision underscores the effectiveness of this intervention.

Despite the rarity of pericatheter cysts, this case suggests that healthcare professionals should maintain a high index of suspicion when treating patients with a ventriculoperitoneal shunt who present with altered neurological status. It is essential to distinguish this condition from other potential causes, such as tumour, or abscess, as its management differs from these causes and carries a favourable prognosis.

## Author contributions

L.S. authored the initial manuscript. B.Z. reviewed the manuscript prior to submission was a senior clinician involved in the patient’s medical care. All authors reviewed and approved the final version of the manuscript prior to submission.

## Conflict of interest statement

L.S. and B.Z. report no conflict of interests to declare.

## Funding

No external funding was received for the preparation of this report. The authors are employees of Northern Sydney Local Health District, where the patient was treated. The authors’ employer was not involved in the production or content of this manuscript, or the decision to publish.

## Data availability

Clinical data related to this case is retained by Northern Sydney Local Health District, NSW, Australia. This data is not available for public release due to legislative requirements regarding patient confidentiality.

## Ethics and consent for publication

Executive approval for this case report was obtained from the Northern Sydney Local Health District Human Research Ethics Committee prior to submission, in accordance with local ethics regulations.

Informed written consent was provided by the patient’s next of kin (acting as her person responsible) for publication of this case. A copy of the consent form is retained by the authors and will be provided to the Editor-In-Chief upon request.
